# Who are the “police” in “police violence”? Fatal violence by U.S. law enforcement agencies across levels of government

**DOI:** 10.1186/s40621-024-00496-3

**Published:** 2024-04-04

**Authors:** Jaquelyn L. Jahn, Gabriel L. Schwartz

**Affiliations:** 1 Department of Epidemiology and Biostatistics, The Ubuntu Center on Racism, Global Movements and Population Health Equity, Drexel University Dornsife School of Public Health, 3600 Market St., Philadelphia, PA 19104 USA; 2 Department of Health Management and Policy, Urban Health Collaborative, Drexel University Dornsife School of Public Health, 3600 Market St., Philadelphia, PA 19104 USA

**Keywords:** Police violence, Health equity, Law enforcement

## Abstract

**Background:**

Police violence is increasingly recognized as an urgent public health problem. Basic questions about police violence, however, remain unanswered, including which types of law enforcement agency are responsible for fatal police violence deaths.

**Methods:**

We estimated the proportion of police violence deaths in the U.S. (2013–2022) that were attributable to local, county, state, federal, or tribal police agencies, using mapping police violence data. We examined proportions overall, by decedent race/ethnicity, and by state.

**Results:**

Nationally, 60% of decedents were killed by municipal, 29% by county, 8% by state, and 3% by federal, police, with < 1% killed by tribal or other officers. These proportions varied by race/ethnicity, with 56% of Native American decedents killed by municipal police compared to 70–75% among other racially minoritized people. While municipal police were responsible for most deaths in most states, in the Southeast, county police predominated. In some Northeastern states (and Alaska), state police were responsible for > 40% of deaths.

**Conclusions:**

We identify wide geographic & racial/ethnic variation in the agencies responsible for fatal police violence. Findings suggest that the budgetary and infrastructural shifts required to prevent fatal police violence need to occur at multiple levels of government.

**Supplementary Information:**

The online version contains supplementary material available at 10.1186/s40621-024-00496-3.

## Background

Over the last decade, political movements led by Black Americans have drawn increased attention to police violence (Alang et al. 2017). Killings by law enforcement officers and racial inequities therein have become a point of contention for policymakers as affected communities call for accountability and justice. Yet public health and social science research on who, exactly, is doing the injuring remains sparse. Research is currently unclear, for example, on whether fatal police violence is primarily driven by local municipal police, county police, state police, or federal law enforcement officers.

For accountability and prevention, differentiating between these agencies is critical, as each is regulated by different levels of government and is thus accountable to different legislative, electoral, and non-electoral processes (O’Rourke et al. 2021; Cook and Fortunato 2023). The budgets and funding mechanisms supporting these agencies are also distinct; efforts to shift “police” spending are contingent on where different agencies’ funding comes from. Moreover, hierarchies of law enforcement jurisdictional authority can differ across states and regions, as do the roles of different law enforcement agencies (Moore 1997). In Alaska, for example, county sheriffs do not exist, nor do counties; equivalent functions are carried out by Alaska State Troopers (Hopkins 2019). Connecticut similarly considers sheriffs to be state employees, though their duties are governed by local governments (Hoffman 2000). In states such as Arizona or South Dakota, American Indian reservations cover large swaths of land (Indian Lands of Federally Recognized Tribes of the United States 2014), areas in which tribal police, local municipal police, state police, and federal officers from the Bureau of Indian Affairs may each have either sole or overlapping jurisdiction, depending on the crime, state, exact location, and tribal membership of both the victim and the perpetrator of a crime (Mantegani 2021).

Investigating which types of law enforcement agency are responsible for the broader problem of fatal police violence is difficult via official government data, in part because of inconsistent data collection requirements across agencies. Reporting deaths during detention, arrest, or while in custody of federal officers is explicitly required by the Death in Custody Reporting Act (DCRA) of 2013 (P.L. 113–242). Working with individual-level data on these deaths, however, is restricted by federal officials and is only allowed in protected data centers (United States. Bureau of Justice Statistics 2020). In contrast, deaths by local municipal police departments are only collected if these data are voluntarily reported by local agencies to the federal Bureau of Justice Statistics (BJS), or if the collection and dissemination of this data is required by individual court orders or by state, county, or municipal legislation. Meanwhile, at the state level, deaths by state officers are not required to be reported to the federal government at all; data collection for these deaths is instead governed by widely varying state policies (Krieger et al. 2015). As a result, neither the public nor policymakers have any federally-collected comprehensive data on which types of law enforcement agency are killing civilians.

The objective of this paper is thus to provide descriptive information on counts and rates of people killed by law enforcement officers across different levels of government in the United States. We further examine state-level heterogeneity in terms of how these deaths are distributed across agencies. We do so by analyzing 10 years of data from Mapping Police Violence (MPV), a community science initiative that gathers and validates data from both official sources and the media on incidents of fatal violence by law enforcement officers—including which agencies were responsible for each death. Our analysis provides information for public health workers and the general public to effectively demand democratic accountability, prevent fatal police violence, and protect our collective health.

## Methods

### Study population

Since 2013, Mapping Police Violence (MPV) has generated a near-comprehensive database of fatal police violence in the U.S. by sourcing information from police and media reports. Cases are reviewed by two sets of researchers to ensure accuracy (Campaign Zero 2022). Data are further cross-referenced with a database maintained by *The Washington Post* to validate information and detect discrepancies. MPV researchers additionally conduct follow-up internet searches to adjudicate discrepancies between sources and fill in missing information about decedents (e.g., searching through social media, police reports, and obituaries to determine decedents’ race/ethnicity).

Importantly, MPV defines fatal police violence as “any incident where a law enforcement officer (off-duty or on-duty) applies, on a civilian, lethal force resulting in the civilian being killed whether it is considered ‘justified’ or ‘unjustified’ by the U.S. Criminal Legal System” (Campaign Zero 2022). MPV thus excludes incidents reported to be caused by speeding or crashing during a police chase, overdose, and in most cases, jumping from a height in a foot chase—i.e., cases in which reviewers determined that police did not directly apply lethal force. Over our study period of January 1, 2013 to December 31, 2022 (from the first year these data were gathered prospectively, in 2013, to the most recently available data at the time of download) there were 11,189 incidents of fatal police violence in the database; 39 incidents lacked information on agency, leaving a total of 11,150 deaths in our dataset.

### Agency classification

The agency of the officer is manually entered by MPV researchers. For instances when officer agency differs from the jurisdiction in which the incident took place (including off-duty incidents), the officer agency is used. We iteratively classified MPV-entered agencies into mutually exclusive categories by identifying key words in agency names, manually reviewing the results of our initial categorization, conducting additional research about specific cases and agencies where necessary, and recoding agencies that we had miscategorized. Manual review was conducted separately by each author, after which cases that either author flagged as ‘unclear’ were reviewed jointly before making a final determination. Our categories included federal, tribal, state, local (county and non-county), university (university police forces), and school (police officers or security in K-12 schools). For deaths in which multiple agencies were responsible, we separately classified each agency. (See Additional file 1: Table S1 for a detailed list of search terms.)

### Statistical analyses

We first examined counts and percentages of deaths for each governmental level of law enforcement agency, overall and stratified by decedent racialized group. Next, we calculated counts and percentages of deaths attributed to each agency level for each state and mapped these estimates. We then calculated state-level rates of fatal police violence attributed to different law enforcement agencies by dividing counts by state population using data from the American Community Survey 5-year estimates (2013–2019, 2021–2022) and U.S. decennial census (2020) and multiplying by 100,000.

### Sensitivity analyses

Lastly, we examined whether state-level differences in rates were entirely explained by differences in the size of each state’s various law enforcement agencies at different levels of government. That is, for example: do states with a high proportion of deaths attributable to state officers really have a more lethal state police force? Or does that state simply employ more state police officers?

To do so, we used data on the number of full-time sworn officers from the Law Enforcement Management and Administrative Statistics (LEMAS) survey. Conducted periodically since the 1980s, LEMAS collects data on roughly 3000 law enforcement agencies at the state, county, and local levels, including all large agencies (those with 100 or more employees) as well as a sample of smaller agencies meant to be nationally representative (with accompanying survey weights). To align with our study period, we used LEMAS data for the years 2013, 2016, and 2020. (Washington D.C., included as a state in our analysis, was excluded from LEMAS 2013 data; we thus exclude D.C.’s data for 2013–2015). We applied survey weights and carried forward 2013 estimates through 2015, 2016 estimates through 2019, and 2020 estimates through 2022. The LEMAS data for these years groups together local, county, and regional police departments; we combined these with Sheriffs' offices to be consistent with our coding of agencies in the fatality data.

We conducted our sensitivity analyses in two ways, focusing on (A) state police and (B) local/county police (including sheriffs). First, for each state, we divided the number of deaths attributable to a given type of agency by the state’s population, multiplied by 100,000. We then divided by the total number of officers in that state for that type of agency. (For example: divide deaths attributable to state police by the state’s population, multiply by 100,000, and then divide by the number of state police in that state.) This metric represented the average number of people killed *per officer* in each state, for both local/county officers and state officers.

Secondary sensitivity analyses approached the issue of different states having different numbers of officers for a given agency type in a different way. These analyses used multilevel Poisson models. Such models analyzed state-year data, with state-years clustered within states. These models separately estimated the number of deaths for a given agency type per capita (i.e., the outcome was a count of deaths for that agency type in that state-year, with the population of that state in that year included as an offset). In essence, we first estimated a null multilevel model with random effects for states, then estimated a second multilevel model that adjusted for the number of full-time officers per capita, then compared whether states with high state-level residuals from the first null model also had high state-level residuals from models that controlled for the number of officers per capita in that state.

## Results

Nationally, across the 11,150 incidents of fatal police violence in our data, most were attributed to officers from local law enforcement agencies, including around 30% (n = 7073) involving local county agencies and, separately, 60% involving local non-county agencies (n = 3460)—i.e., largely municipal police departments. This pattern held across incidents involving only one agency as well as those involving multiple agencies (5% of total; see Table 1). Around 8% of deaths involved state law enforcement agencies and about 3% involved federal agencies.

**Table 1:** Law enforcement-related deaths by agency level 2013–2022 in mapping police violence

Agency	Single agency deaths (*N* = 10,550)	Multiple agency* deaths (*N* = 600)	Total deaths (%) (*N* = 11,150)
Federal	261	68	329 (2.8%)
Tribal	28	9	37 (0.31%)
State	715	227	942 (7.9%)
Local			
Municipal	6508	563	7073 (60%)
County	3008	452	3460 (29%)
University	28	6	34 (0.29%)
School	2	0	2 (0.02%)

*5% of the deaths in this database included multiple agencies. For these deaths, we double or triple counted the death to show all of the involved agencies. Therefore, the counts in the multiple agency deaths column add up to more than the total of 600

### Racial and ethnic variation

In every racialized group, most single-agency incidents were attributed to local agencies, with the majority committed by municipal officers (Table 2). Yet there remained substantial variation—particularly comparing Native Americans to other racially and ethnically minoritized groups. Whereas municipal agencies committed 69–75% of fatal police violence against other racially/ethnically minoritized groups, municipal agencies committed only 56% of fatal police violence against Native American people. This was driven by a greater proportion of Native American decedents who were killed by tribal agencies (8.6% [n = 12], compared to < 1% for other groups), federal agencies (6.4%, compared to 2–3% for other minoritized groups), and state agencies (9.3%, compared to 3–4% for other minoritized groups).

**Table 2:** Law enforcement-related deaths involving one agency by decedent race and ethnicity (2013–2022)

Agency	Asian (*N* = 153)	Black (*N* = 2712)	Hispanic (*N* = 1888)	Native American (*N* = 140)	Native Hawaiian and Pacific Islander (*N* = 61)	Unknown race (*N* = 1093)	White (*N* = 4503)
Federal	4 (2.6%)	66 (2.4%)	57 (3.0%)	9 (6.4%)	2 (3.3%)	29 (2.7%)	94 (2.1%)
Tribal	0 (0%)	1 (< 0.1%)	3 (0.2%)	12 (8.6%)	0 (0%)	8 (0.7%)	4 (0.1%)
State	5 (3.3%)	119 (4.4%)	79 (4.2%)	13 (9.3%)	4 (6.6%)	86 (7.9%)	409 (9.1%)
Local							
Municipal	106 (69.3%)	1952 (72.0%)	1328 (70.3%)	79 (56.4%)	46 (75.4%)	608 (56.0%)	2389 (53.0%)
County	37 (24.2%)	562 (20.7%)	415 (22.0%)	27 (19.3%)	9 (14.8%)	360 (32.9%)	1598 (35.5%)
University	1 (0.7%)	12 (0.4%)	5 (0.3%)	0 (0%)	0 (0%)	2 (0.2%)	8 (0.2%)
School	0 (0%)	0 (0%)	1 (0.1%)	0 (0%)	0 (0%)	0 (0%)	1 (< 0.1%)

Non-Hispanic White decedents were also less likely to be killed by municipal agencies, at only 53%, relative to proportions among Asian, Black, and Hispanic decedents (Table 2). This lower municipal proportion for White decedents appeared driven by heightened proportions committed by state police and, uniquely, a higher proportion committed by county police (35%, compared to 15–24% for other racialized groups).

### Geographic variation

**Fig. 1:**
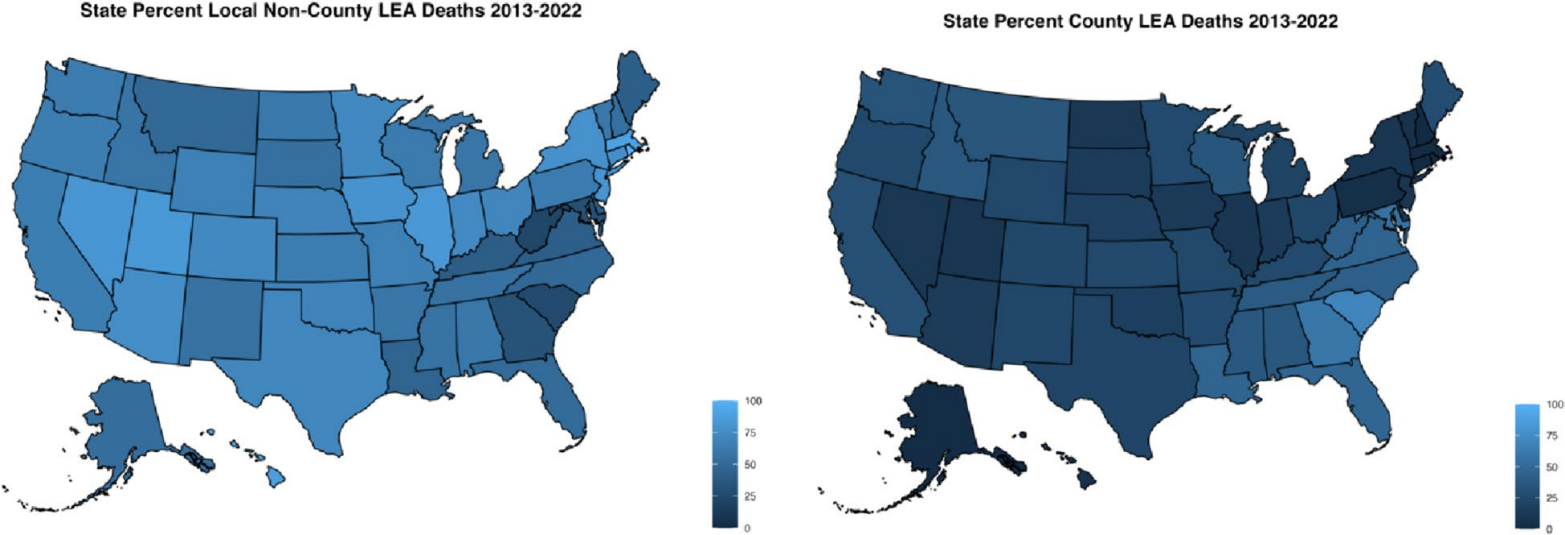
Percent of deaths involving local non-county and county law enforcement agencies in each state, 2013–2022. *Note: Displayed percentages were calculated only using deaths where a single agency was responsible. Data on decedents are from the Mapping Police Violence database

There were also geographic differences in the law enforcement agencies responsible for these deaths across states, although in most states local municipal law enforcement was involved in the majority (Fig. 1). Maps and tables of the count and rate of deaths in each state attributable to each agency type are available in Additional file 1: Fig. S1 and S2, as well as Additional file 1: Table S2.

Most fatal police violence committed by federal agencies occurred in Texas (n = 36), Arizona (n = 23) and California (n = 22), but these represented a small proportion of the fatal police violence overall in those states (3.67%, 4.82%, and 1.38% respectively); further, when scaled by state population, these states did not have the highest rates of federal law enforcement fatal violence (rates were 0.01, 0.03, and 0.01 per 100,000 respectively). The Dakotas, however, were clear outliers, with federal agencies responsible for 22% (n = 4) of fatal police violence deaths in North Dakota (a rate of 0.05 deaths per 100,000 state population) and 11% (n = 4) in South Dakota (a rate of 0.05 deaths per 100,000), patterns which held when examined relative to their populations. Relative to state population, rates by federal law enforcement in New Mexico were also high, at 0.07 deaths per 100,000 state population (n = 14, 6.6% of all fatal police violence in the state). Deaths in New Mexico due to federal law enforcement agencies were largely (n = 8 of 14) due to Department of Justice agencies (e.g., FBI, US Marshals, and Bureau of Alcohol, Tobacco, Firearms & Explosives) and to Homeland Security (n = 5 of 14).

Most fatal police violence committed by tribal agencies occurred in Arizona (n = 7), South Dakota (n = 6) and Oklahoma (n = 5), all states containing large American Indian reservations. As a proportion and as a rate, however, South Dakota was again a clear outlier, with tribal agencies there responsible for 1 in 6 (17%) fatal police violence deaths in that state, a rate of 0.07 deaths per 100,000 population. By contrast, in Arizona, the equivalent proportion was 1.5% (a rate of 0.01 deaths per 100,000).

In a handful of states particularly in the northeast and mid-Atlantic, state law enforcement agencies were involved in over a third of fatal police violence incidents. In New Hampshire, for example, 45% of deaths involving law enforcement were attributed to state agencies (n = 9, rate = 0.07 deaths per 100,000 state population), and in Delaware this percentage was 44% (n = 11, rate = 0.12 deaths per 100,000). Pennsylvania had the highest number of deaths involving state law enforcement (n = 69, 0.05 deaths per 100,000), representing 29.74% of fatal police violence in that state. And outside the northeast, other states emerged has having particularly high rates of fatal violence committed by state law enforcement, including Alaska (0.33 per 100,000), West Virginia (0.15 per 100,000), New Mexico (0.13 per 100,000), and Kentucky (0.12 per 100,000). After scaling these rates by the number of state law enforcement officers, Alaska’s rate remained elevated above other states (Additional file 1: Fig. S3). This also held in our secondary sensitivity analyses: states with unusually high (or low) rates of fatal violence committed by state law enforcement officers in general still appeared to have elevated rates even after controlling for the number of state law enforcement officers per capita (Additional file 1: Fig. S4).

At the local level, in most states local (including county) law enforcement agencies accounted for most of the fatalities. This held after scaling by the number of local law enforcement officers, although Alaska emerged as having a particularly high rate relative to the size of its local law enforcement agencies (Additional file 1: Fig. S5 & S6). When separating local municipal from county law enforcement agencies, the Southeast stood out for the high proportion of deaths involving county law enforcement (Fig. 1). In South Carolina, Georgia, Maryland, Florida, Louisiana, Virginia, West Virginia, and North Carolina, county agencies were involved in over 40% of deaths by law enforcement officers in those states, reaching nearly 70% in South Carolina. Rates attributable to county law enforcement were also high in these states relatively, in addition to New Mexico.

## Conclusions

Protests against fatal police violence—particularly racial inequities in that violence—have erupted across the country in recent years, including in Minneapolis, MN; Ferguson, MO; New York, NY; and Portland, OR. These protests have resulted in notable, if limited, changes to municipal policy and oversight, including a shift of 4.5% of the Minneapolis Police Department’s budget to violence prevention programs (Gross and Eligon 2020) and a series of federal investigations into these cities’ uses of force and adherence to constitutional law (Berman and Lowery 2021; Levinson 2022; Max 2023). Yet equivalent policy attention towards county, state, or federal agencies are comparatively rare, though not without exception (or pushback from law enforcement agencies) (Hedgpeth 2021; Adams 1972; Sheriffs’ Statement on IACP Policy on Use of Force - National Sherriffs' Association 2024).

Our results suggest that for a sizable minority of US states and for specific racially minoritized groups, the focus on municipal police departments has overshadowed agencies at higher levels of government, which in some cases perpetuate close to half of all fatal police violence. This has implications both for the targets of police accountability and policy change, as well as for police violence research.

### Implications for research and advocacy: geography matters

Our results are clear that where one lives in the United States has major implications for how residents are policed, who is policing them, and what mortal risks they encounter when interacting with officers of different law enforcement agencies. Collaborations between quantitative epidemiologists and sociologists, historians, political scientists, and economists are necessary to better understand this state-level heterogeneity, including richer dissections of what drives state reliance on state versus county versus local law enforcement, as well as how these determinants have shifted over time. State heterogeneity may be reflective of the differing social control functions of different levels of government across place. Previous research has largely focused on characteristics of cities (Beck and Goldstein 2018) or of specific states (Gilmore 2007; Santo and Dunlop 2021), including the role of city politics and policy, municipal police force size, and budgetary decisions as determinants of criminalization and fatal police violence. Less research has focused on the political economy of relying on federal, state, county, or municipal officers for policing (particularly, policing that is likely to result in a civilian’s death), nor on how shifting policing responsibilities from officers at one level of government to another may elide local democratic accountability. Our analysis suggests that the role of county police, particularly in the U.S. South, and state police in New Hampshire, Pennsylvania, Delaware, and Alaska, may be particularly deserving of further investigation and action.

However, research on policing and the delegation of police responsibilities across different agencies is severely hindered by available data. The state-level heterogeneity we observe may partially reflect differences in how states fund and distribute patrolling officers across agencies at different levels, or it may reflect differences in the accountability mechanisms officers face at different levels of government in different states; but the data needed to adjudicate between those theories does not exist. Complicating the utility of our LEMAS analysis, roughly 1 in 5 local and county law enforcement agencies choose not to participate in that main, federal survey where law enforcement personnel data is collected (Davis and Goodison 2020). Moreover, available budgetary data from the U.S. Census of Governments includes amounts spent on law enforcement at different levels of government, but detailed knowledge about each state’s funding structure would be required to tell whether, e.g., state spending on policing that is transferred to the local level is (A) used to fund state officers whose pay is simply administered by local governments, (B) used for various law enforcement-related programs at the discretion of local governments, or (C) used by local governments lacking their own police departments to contract with county-level officers.

### Implications for research and advocacy: racialized bureaucracies of state violence

Equally important are the racialized dynamics of place-specific state power in perpetuating fatal police violence. Native American scholarship has brought clear attention to the ways accountability for violence against Native Americans is undermined by what is frequently referred to as a “jurisdictional maze” (Mantegani 2021; Mendoza 2020). Unlike other US Americans, violence against indigenous people on reservations (committed by either police or civilians) may fall under simultaneous or exclusive jurisdiction of federal, state, or tribal agencies, depending on the state, type of violence, and tribal membership of both the victim and the perpetrator (Perry 2009). This jurisdictional maze may mean that anti-indigenous police violence, driven by what Beardall describes as “sovereignty threat” (Beardall 2021), is less subject to legal or democratic accountability, such that indigenous people in states with large reservations may be both more targeted by the police and less likely to be able to hold law enforcement officers accountable for that violence (Perry 2009, 2006).

Native American activists have accordingly called not only for an end to US state violence against their peoples but also for policy changes that would return judicial and prosecutorial power to tribes. Most famously, in 1972, Native leaders occupied the Bureau of Indian Affairs (BIA) in DC (Hedgpeth 2021), demanding (A) the abolition of the BIA (who were given concurrent jurisdiction over all “major crimes” committed on reservations by the Major Crimes Act of 1885; tribes’ authority to prosecute non-Native perpetrators for any crime was subsequently revoked by the Supreme Court in their 1978 decision in *Oliphant vs. Suquamish Indian Tribe*) and (B) the repeal of state laws enacted under Public Law 280, federal legislation that required certain states (and allowed other states) to adopt jurisdiction over criminal offenses on reservation lands (Mantegani 2021; Adams 1972; Mendoza 2020).

Epidemiologists and other social scientists must bring similarly historically-informed analyses to bear when investigating drivers of fatal police violence by specific agencies against specific racialized groups—proceeding in their research with the understanding that the US government has constructed specific bureaucracies of state violence in their efforts to exert racist control against different peoples of color. Calls for change must similarly engage with the racial specificity of those social control regimes.

### Limitations and strengths

Our study has important strengths and limitations. On the one hand, we analyze 10 years of the best available data on fatal police violence in the US, collected as comprehensively as possible (Feldman and Bassett 2021) and with rigorous fact-checking. Our data are also novel, showing remarkable geographic and racial/ethnic heterogeneity in who is responsible for fatal police violence. On the other hand, roughly 10% of our sample was missing information on decedents’ race/ethnicity, and a handful of cases (n = 39) were excluded because they were missing a responsible agency, potentially biasing the proportion of each group’s deaths attributable to different agencies. Despite its relative comprehensiveness, MPV almost certainly missed at least some cases of fatal police violence, with an unknown distribution of those cases across agencies and racial/ethnic groups. Results should thus be interpreted with appropriate caution.

## Conclusions

Our research identifies the different levels of government responsible for fatal police violence across US states and racialized groups, with implications for preventing deaths perpetuated by law enforcement. Abolitionists have argued that the need for police (and thus for police violence) would be obviated if public funds were instead spent on community-led prevention measures such as anti-poverty programs, a proactive right to housing, universal health care (including mental health care), safer gun laws, and unarmed crisis intervention teams (Conner et al. 2021; Kaba and Ritchie 2022). Others have called for accountability in the form of organizational reforms to existing law enforcement agencies, including to officer training, use-of-force policies, qualified immunity, and prosecution of officers who injure civilians (Weisburd et al. 2022; Gross 2017). Our findings suggest that the budgetary and infrastructural shifts required to prevent fatal police violence need to occur at multiple levels of government, and that the specific agencies accountable also differ across place. We hope this paper can guide community organizations, epidemiologists, and collaborations between the two in their efforts to prevent fatal violence by law enforcement officers and thereby safeguard population health.

### Supplementary Information

**Additional file 1**. Supplemental Methodology and Results.

## Data Availability

Mapping Police Violence data is publicly available and free to download at: mappingpoliceviolence.org.
