# Supplementary material for: Who are the “police” in “police violence”? Fatal violence by U.S. law enforcement agencies across levels of government
Source: Inj Epidemiol. 2024 Apr 4;11:13. doi: 10.1186/s40621-024-00496-3 (PMC10993425; doi:10.1186/s40621-024-00496-3)
Supplement: Supplementary file 1 — Additional file 1. Supplemental Methodology and Results. [file 40621_2024_496_MOESM1_ESM.docx]

**Supplemental Material**

**Supplemental** **Table 1.** Coding scheme for classifying law enforcement agency levels

| **Agency classification** | **Key words*** | **Example exceptions**  **(recoded out of this category after review)** |
| --- | --- | --- |
| Federal | “u.s.”; “immigration”; “federal”; “bureau”; “border”; “fugitive”; “capitol police”; “ us homeland security”; “us marshals”; “secret service”’; “metro fugitive task force”; “lone star fugitive task force”; “us park police” | “federal heights police department”; “harrisburg police bureau” |
| Federal: Department of Defense | “air force”, “defense”, “pentagon”, “navy”, “marine” | N/A |
| Federal: Department of Homeland Security | “border”, “immigration”, “homeland”, “federal protective service”, “secret service” | N/A |
| Federal: Department of Justice | “alcohol”, “drug”, “marshal”, “bureau of investigation”, “narcotics”, “fugitive” | N/A |
| Federal: Federal Bureau of Investigation | “bureau of investigation” | N/A |
| Federal: Federal Marshals Service | “marshal”; “fugitive” | N/A |
| Federal: Department of the Interior | “Indian”, “park”, “land” | N/A |
| Federal: Department of Veterans Affairs | “veterans” | N/A |
| Tribal | “tribe”; “rancheria”; “reservation”; “blackfeet police department”; “Cherokee nation marshal service”; “Choctaw police department”; "pueblo of laguna police department";"taos pueblo department of public safety";"hualapai nation police department";"white mountain apache police department" | N/A |
| State | State names; “rail”; "port authority police","dc metropolitan police department","dc metro transit police","special police washington dc" | “colorado springs crisis response team”; “new york police department” |
| Local (including county and non-county) | “county”; “parish”; “police”; “city”; “metropolitan”; ”township”; “public safety”; “sheriff”; “cleveland metroparks ranger department", "detroit transportation corporation", "downriver swat team", "eau claire s.w.a.t", "port arthur pollice department","colorado springs crisis response team" | N/A |
| *Local (county)* | “county”; “parish”; "prince george police department","prince george's police department" | N/A |
| *Local (non-county)* | Local excluding those classified as county | N/A |
| University | “university”; “college”; “georgia tech police department”; "coppin state police department" | "university heights police department"; "college station police department" |
| School | “school”; “district” | "regional transportation district transit police" |

* Note: Key words include initial search terms as well as strings that were added to correctly classify specific cases after coding reconciliation across the two authors. State park police (e.g. “Illinois department of natural resources conservation police”) are coded as state-level agencies. Railroad police are counted as state law enforcement.

**Supplemental Figure 1.** Percent of deaths involving different types of law enforcement agencies in each state, 2013-2022


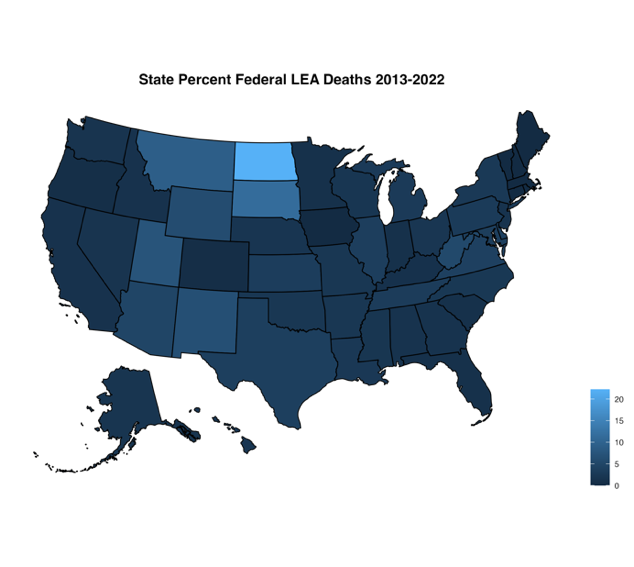
**
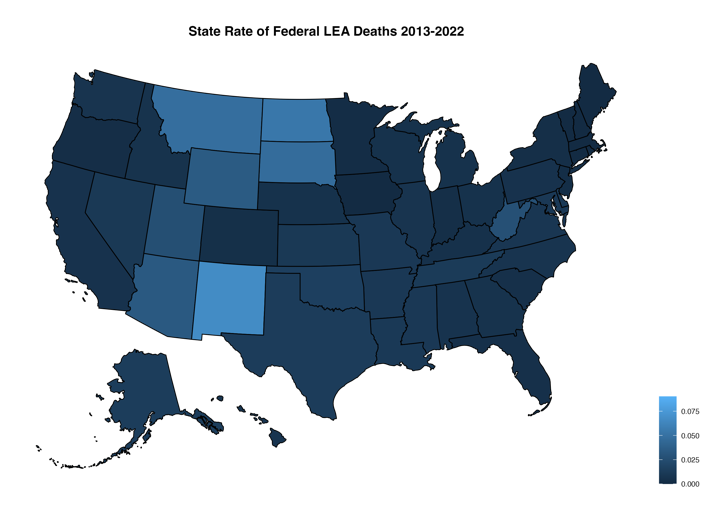
**

**
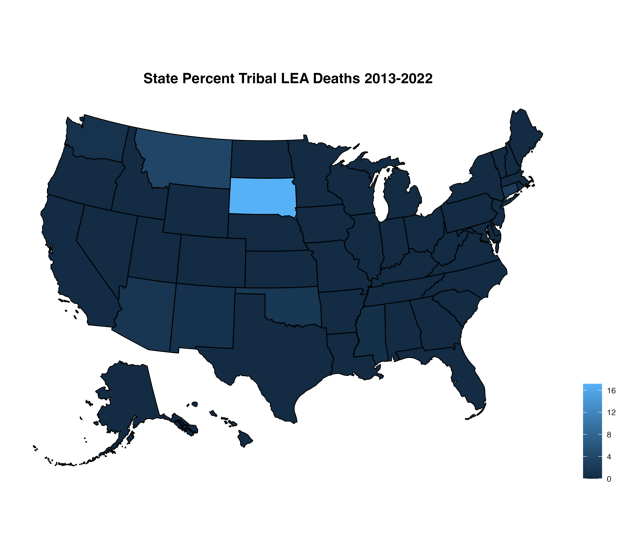

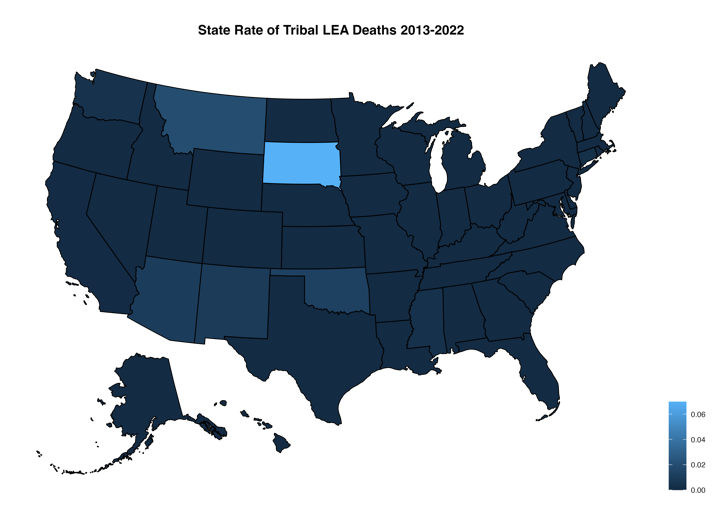
**

**
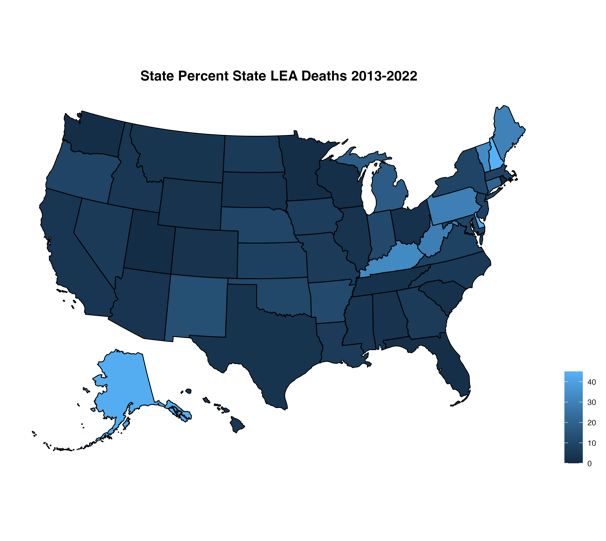

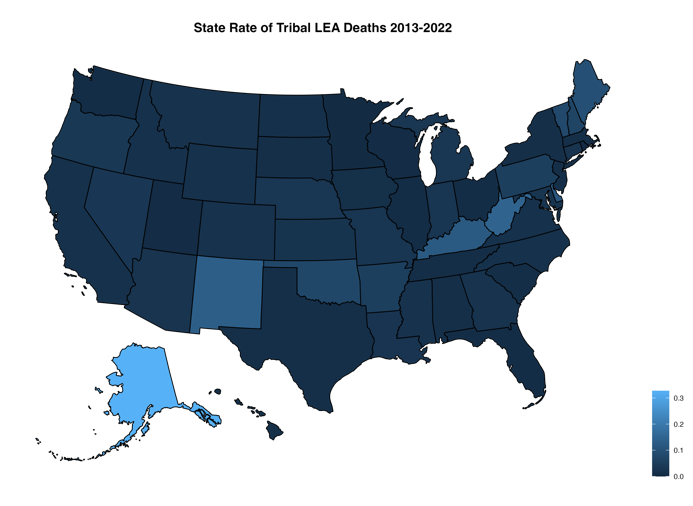
**

**
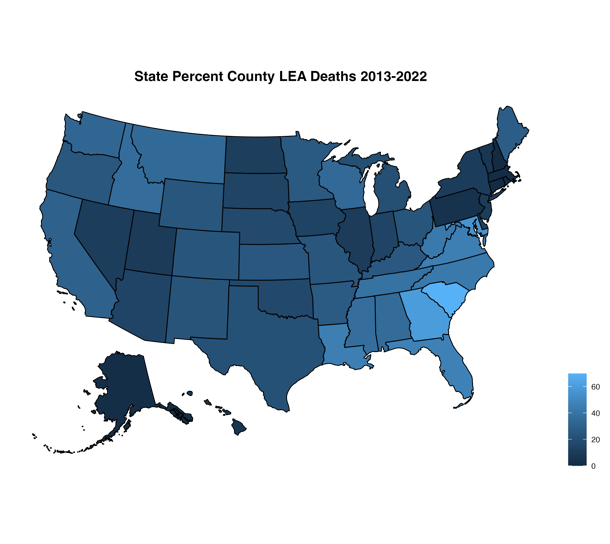

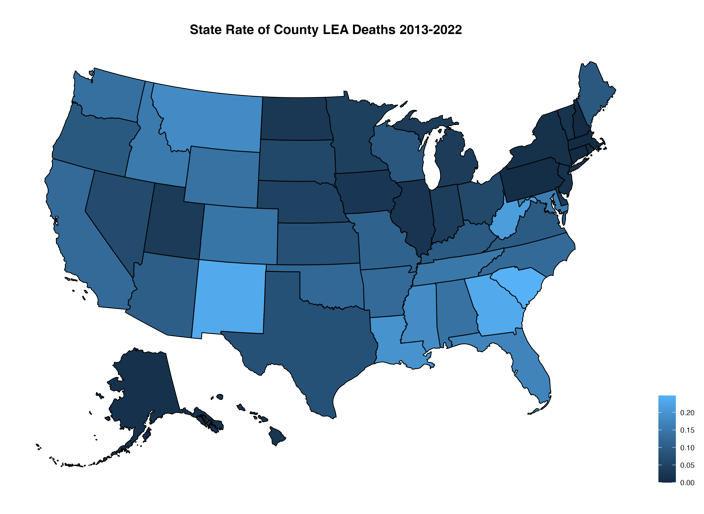
**

**
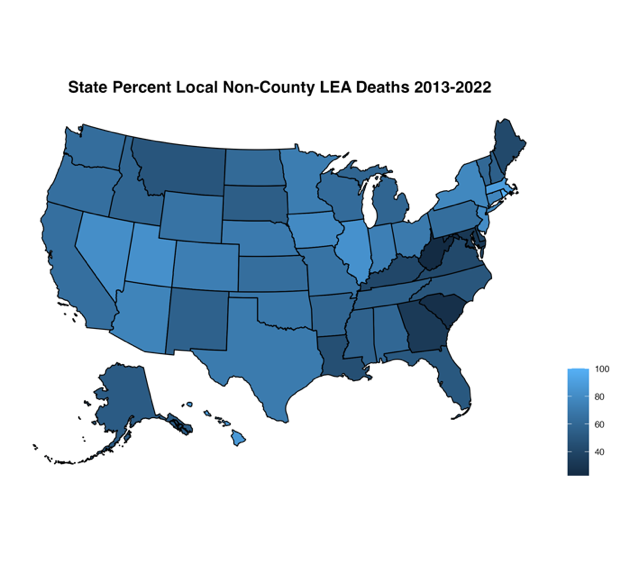

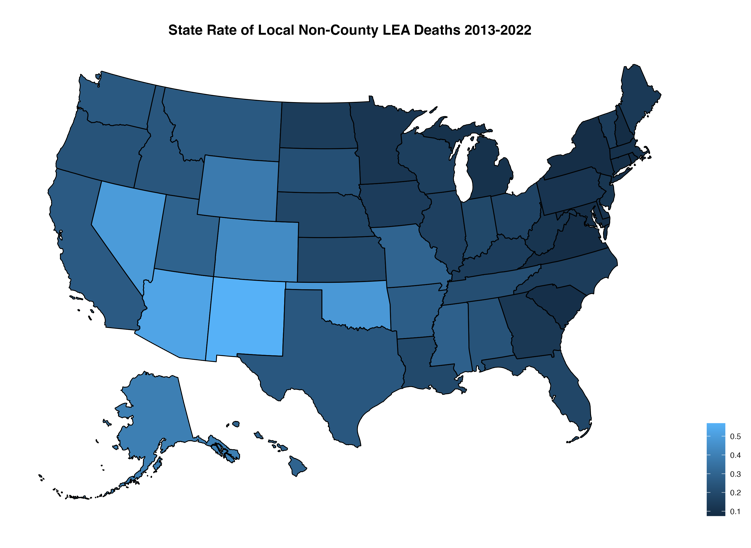
**

* Note: Displayed percentages were calculated only using deaths where a single agency was responsible (the numerator in the column to the right). Data on decedents are from the Mapping Police Violence database. Denominator data for the right-most column are derived from the American Community Survey and 2020 decennial US Census.

**Supplemental Figure 2**. Number of deaths in each state attributable to different agency types in the Mapping Police Violence database, 2013-2022


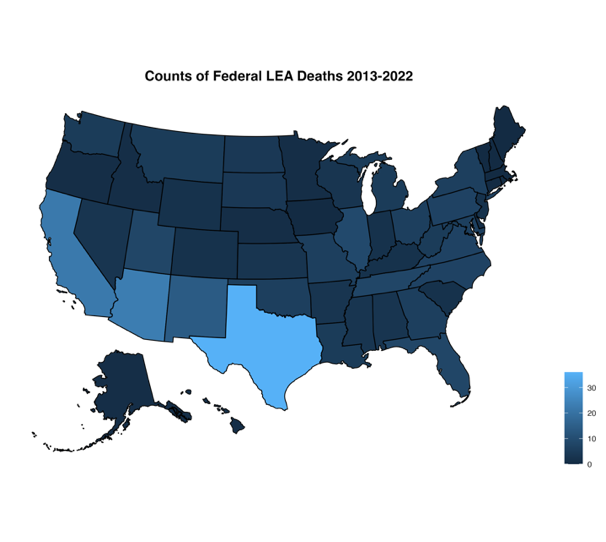
**
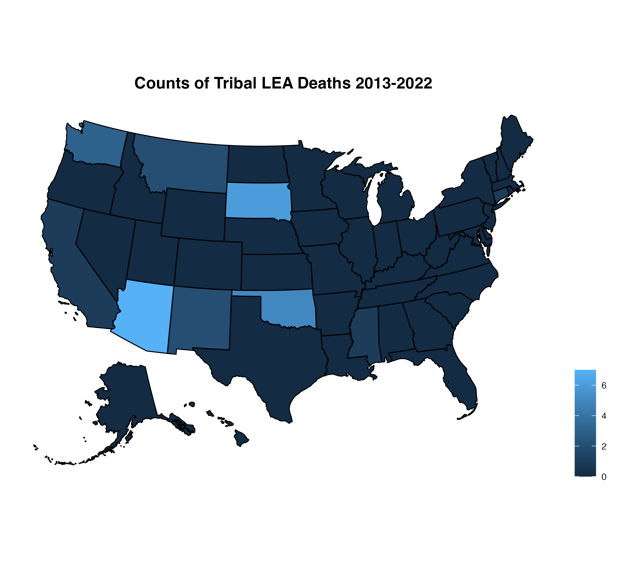

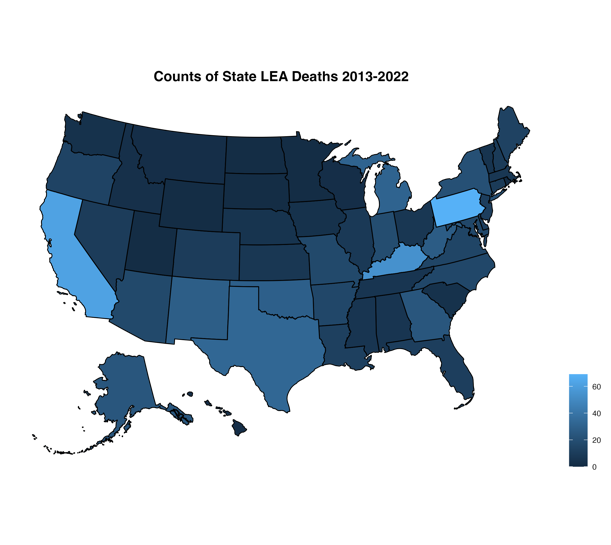

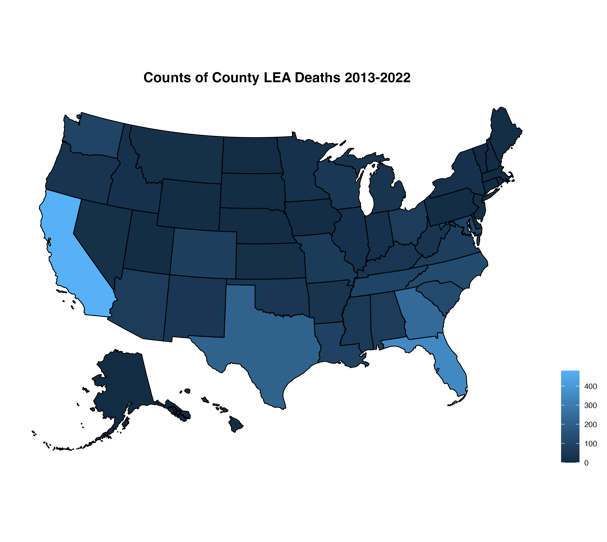

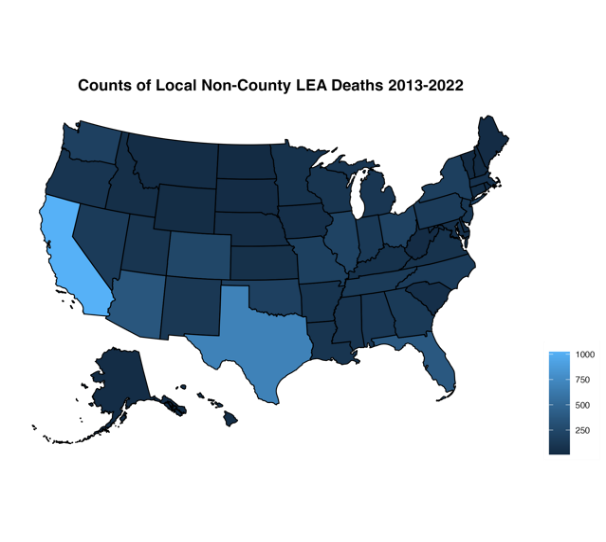
**

* Note: Displayed counts were tabulated only using deaths where a single agency was responsible. Data on decedents are from the Mapping Police Violence database.

**Supplemental Table 2.** Number and percentage of fatal police violence deaths attributable to different agencies, by state (2013-2022)

| **State** | **Number of fatal police violence deaths attributable to agencies at different levels** | | | | | | | | | **% of fatal police violence deaths attributable to agencies at different levels** | | | | | | | |
| --- | --- | --- | --- | --- | --- | --- | --- | --- | --- | --- | --- | --- | --- | --- | --- | --- | --- |
|  | *Fed.* | *Tribal* | *State* | *Local* | *Local (Non-County)* | *Local (County)* | *Univ.* | *School* | *Total* | *Fed. %* | *Tribal %* | *State %* | *Local %* | *Local (Non-County) %* | *Local (County) %* | *Univ. %* | *School %* |
| AK | 1 | 0 | 24 | 30 | 29 | 1 | 0 | 0 | 55 | 1.82 | 0.00 | 43.64 | 54.55 | 52.73 | 1.82 | 0.00 | 0.00 |
| AL | 3 | 0 | 6 | 183 | 115 | 68 | 0 | 0 | 192 | 1.56 | 0.00 | 3.13 | 95.31 | 59.90 | 35.42 | 0.00 | 0.00 |
| AR | 3 | 0 | 16 | 119 | 82 | 37 | 1 | 0 | 139 | 2.16 | 0.00 | 11.51 | 85.61 | 58.99 | 26.62 | 0.72 | 0.00 |
| AZ | 23 | 7 | 17 | 430 | 361 | 69 | 0 | 0 | 477 | 4.82 | 1.47 | 3.56 | 90.15 | 75.68 | 14.47 | 0.00 | 0.00 |
| CA | 22 | 1 | 62 | 1504 | 1024 | 480 | 5 | 1 | 1595 | 1.38 | 0.06 | 3.89 | 94.29 | 64.20 | 30.09 | 0.31 | 0.06 |
| CO | 2 | 0 | 11 | 319 | 240 | 79 | 0 | 0 | 332 | 0.60 | 0.00 | 3.31 | 96.08 | 72.29 | 23.80 | 0.00 | 0.00 |
| CT | 0 | 1 | 9 | 33 | 33 | 0 | 0 | 0 | 43 | 0.00 | 2.33 | 20.93 | 76.74 | 76.74 | 0.00 | 0.00 | 0.00 |
| DC | 6 | 0 | 2 | 32 | 30 | 2 | 0 | 0 | 40 | 15.00 | 0.00 | 5.00 | 80.00 | 75.00 | 5.00 | 0.00 | 0.00 |
| DE | 1 | 0 | 11 | 13 | 10 | 3 | 0 | 0 | 25 | 4.00 | 0.00 | 44.00 | 52.00 | 40.00 | 12.00 | 0.00 | 0.00 |
| FL | 8 | 0 | 11 | 725 | 377 | 348 | 1 | 0 | 745 | 1.07 | 0.00 | 1.48 | 97.32 | 50.60 | 46.71 | 0.13 | 0.00 |
| GA | 6 | 0 | 24 | 373 | 132 | 241 | 2 | 0 | 405 | 1.48 | 0.00 | 5.93 | 92.10 | 32.59 | 59.51 | 0.49 | 0.00 |
| HI | 1 | 0 | 2 | 45 | 42 | 3 | 0 | 0 | 48 | 2.08 | 0.00 | 4.17 | 93.75 | 87.50 | 6.25 | 0.00 | 0.00 |
| IA | 0 | 0 | 4 | 54 | 46 | 8 | 0 | 0 | 58 | 0.00 | 0.00 | 6.90 | 93.10 | 79.31 | 13.79 | 0.00 | 0.00 |
| ID | 1 | 0 | 3 | 68 | 42 | 26 | 0 | 0 | 72 | 1.39 | 0.00 | 4.17 | 94.44 | 58.33 | 36.11 | 0.00 | 0.00 |
| IL | 9 | 0 | 8 | 233 | 208 | 25 | 0 | 0 | 250 | 3.60 | 0.00 | 3.20 | 93.20 | 83.20 | 10.00 | 0.00 | 0.00 |
| IN | 2 | 0 | 19 | 152 | 127 | 25 | 0 | 0 | 173 | 1.16 | 0.00 | 10.98 | 87.86 | 73.41 | 14.45 | 0.00 | 0.00 |
| KS | 3 | 0 | 7 | 78 | 56 | 22 | 0 | 0 | 88 | 3.41 | 0.00 | 7.95 | 88.64 | 63.64 | 25.00 | 0.00 | 0.00 |
| KY | 2 | 0 | 54 | 109 | 67 | 42 | 0 | 0 | 165 | 1.21 | 0.00 | 32.73 | 66.06 | 40.61 | 25.45 | 0.00 | 0.00 |
| LA | 5 | 0 | 12 | 182 | 91 | 91 | 2 | 0 | 201 | 2.49 | 0.00 | 5.97 | 90.55 | 45.27 | 45.27 | 1.00 | 0.00 |
| MA | 0 | 0 | 7 | 70 | 69 | 1 | 0 | 0 | 77 | 0.00 | 0.00 | 9.09 | 90.91 | 89.61 | 1.30 | 0.00 | 0.00 |
| MD | 6 | 0 | 16 | 148 | 53 | 95 | 1 | 0 | 171 | 3.51 | 0.00 | 9.36 | 86.55 | 30.99 | 55.56 | 0.58 | 0.00 |
| ME | 0 | 0 | 13 | 30 | 18 | 12 | 0 | 0 | 43 | 0.00 | 0.00 | 30.23 | 69.77 | 41.86 | 27.91 | 0.00 | 0.00 |
| MI | 5 | 0 | 31 | 139 | 103 | 36 | 1 | 0 | 176 | 2.84 | 0.00 | 17.61 | 78.98 | 58.52 | 20.45 | 0.57 | 0.00 |
| MN | 1 | 0 | 1 | 91 | 67 | 24 | 0 | 0 | 93 | 1.08 | 0.00 | 1.08 | 97.85 | 72.04 | 25.81 | 0.00 | 0.00 |
| MO | 6 | 0 | 17 | 250 | 183 | 67 | 2 | 0 | 275 | 2.18 | 0.00 | 6.18 | 90.91 | 66.55 | 24.36 | 0.73 | 0.00 |
| MS | 3 | 1 | 6 | 140 | 85 | 55 | 0 | 0 | 150 | 2.00 | 0.67 | 4.00 | 93.33 | 56.67 | 36.67 | 0.00 | 0.00 |
| MT | 5 | 2 | 2 | 46 | 27 | 19 | 0 | 0 | 55 | 9.09 | 3.64 | 3.64 | 83.64 | 49.09 | 34.55 | 0.00 | 0.00 |
| NC | 7 | 0 | 16 | 271 | 146 | 125 | 1 | 0 | 295 | 2.37 | 0.00 | 5.42 | 91.86 | 49.49 | 42.37 | 0.34 | 0.00 |
| ND | 4 | 0 | 1 | 13 | 11 | 2 | 0 | 0 | 18 | 22.22 | 0.00 | 5.56 | 72.22 | 61.11 | 11.11 | 0.00 | 0.00 |
| NE | 1 | 0 | 6 | 44 | 35 | 9 | 0 | 0 | 51 | 1.96 | 0.00 | 11.76 | 86.27 | 68.63 | 17.65 | 0.00 | 0.00 |
| NH | 0 | 0 | 9 | 11 | 11 | 0 | 0 | 0 | 20 | 0.00 | 0.00 | 45.00 | 55.00 | 55.00 | 0.00 | 0.00 | 0.00 |
| NJ | 2 | 0 | 11 | 120 | 107 | 13 | 0 | 0 | 133 | 1.50 | 0.00 | 8.27 | 90.23 | 80.45 | 9.77 | 0.00 | 0.00 |
| NM | 14 | 2 | 28 | 168 | 119 | 49 | 0 | 0 | 212 | 6.60 | 0.94 | 13.21 | 79.25 | 56.13 | 23.11 | 0.00 | 0.00 |
| NV | 3 | 0 | 10 | 163 | 144 | 19 | 0 | 1 | 177 | 1.69 | 0.00 | 5.65 | 92.09 | 81.36 | 10.73 | 0.00 | 0.56 |
| NY | 6 | 0 | 21 | 199 | 176 | 23 | 0 | 0 | 226 | 2.65 | 0.00 | 9.29 | 88.05 | 77.88 | 10.18 | 0.00 | 0.00 |
| OH | 6 | 0 | 7 | 273 | 204 | 69 | 4 | 0 | 290 | 2.07 | 0.00 | 2.41 | 94.14 | 70.34 | 23.79 | 1.38 | 0.00 |
| OK | 6 | 5 | 29 | 236 | 189 | 47 | 0 | 0 | 276 | 2.17 | 1.81 | 10.51 | 85.51 | 68.48 | 17.03 | 0.00 | 0.00 |
| OR | 1 | 0 | 14 | 133 | 96 | 37 | 1 | 0 | 149 | 0.67 | 0.00 | 9.40 | 89.26 | 64.43 | 24.83 | 0.67 | 0.00 |
| PA | 7 | 0 | 69 | 156 | 147 | 9 | 0 | 0 | 232 | 3.02 | 0.00 | 29.74 | 67.24 | 63.36 | 3.88 | 0.00 | 0.00 |
| RI | 0 | 0 | 0 | 8 | 8 | 0 | 0 | 0 | 8 | 0.00 | 0.00 | 0.00 | 100.00 | 100.00 | 0.00 | 0.00 | 0.00 |
| SC | 2 | 0 | 4 | 168 | 46 | 122 | 1 | 0 | 175 | 1.14 | 0.00 | 2.29 | 96.00 | 26.29 | 69.71 | 0.57 | 0.00 |
| SD | 4 | 6 | 1 | 24 | 19 | 5 | 0 | 0 | 35 | 11.43 | 17.14 | 2.86 | 68.57 | 54.29 | 14.29 | 0.00 | 0.00 |
| TN | 8 | 0 | 6 | 242 | 143 | 99 | 0 | 0 | 256 | 3.13 | 0.00 | 2.34 | 94.53 | 55.86 | 38.67 | 0.00 | 0.00 |
| TX | 36 | 0 | 33 | 908 | 694 | 214 | 5 | 0 | 982 | 3.67 | 0.00 | 3.36 | 92.46 | 70.67 | 21.79 | 0.51 | 0.00 |
| UT | 8 | 0 | 1 | 100 | 90 | 10 | 0 | 0 | 109 | 7.34 | 0.00 | 0.92 | 91.74 | 82.57 | 9.17 | 0.00 | 0.00 |
| VA | 7 | 0 | 15 | 154 | 74 | 80 | 1 | 0 | 177 | 3.95 | 0.00 | 8.47 | 87.01 | 41.81 | 45.20 | 0.56 | 0.00 |
| VT | 0 | 0 | 5 | 10 | 9 | 1 | 0 | 0 | 15 | 0.00 | 0.00 | 33.33 | 66.67 | 60.00 | 6.67 | 0.00 | 0.00 |
| WA | 5 | 3 | 4 | 286 | 188 | 98 | 0 | 0 | 298 | 1.68 | 1.01 | 1.34 | 95.97 | 63.09 | 32.89 | 0.00 | 0.00 |
| WI | 3 | 0 | 2 | 143 | 92 | 51 | 0 | 0 | 148 | 2.03 | 0.00 | 1.35 | 96.62 | 62.16 | 34.46 | 0.00 | 0.00 |
| WV | 5 | 0 | 27 | 60 | 21 | 39 | 0 | 0 | 92 | 5.43 | 0.00 | 29.35 | 65.22 | 22.83 | 42.39 | 0.00 | 0.00 |
| WY | 2 | 0 | 1 | 30 | 22 | 8 | 0 | 0 | 33 | 6.06 | 0.00 | 3.03 | 90.91 | 66.67 | 24.24 | 0.00 | 0.00 |

**Supplemental Figure 3**. State-level rates of deaths attributable to state law enforcement, generally vs. per officer, 2013-2022

Here we compare rates of fatal violence by state law enforcement officers at the state level (along the X axis) by the same rates divided by the number of state law enforcement officers employed by a given state (along the Y axis). The clear linear pattern demonstrates that states that experienced high levels of violence by state law enforcement officers in general also tended to have high levels of that violence even after accounting for the fact that those states tend to employ a larger number of state law enforcement officers (ρ=0.92).

Note: DC’s data includes only 2016-2022, as it was not included in 2013 LEMAS data. DC’s data for 2013-2015 was thus excluded from this analysis.

**Supplemental Figure 4**. Modeled state-level residuals from models estimating rates of deaths attributable to state law enforcement, adjusting vs. not adjusting for number of state officers per capita, 2013-2022

Here we compare state-level residuals from multilevel Poisson models estimating rates of fatal violence by state law enforcement officers at the state level, from a null model (along the X axis) vs. a model adjusting for the number of state law enforcement officers per capita in each state (along the Y axis). States that experienced high levels of violence by state law enforcement officers in general also tended to have high levels of that violence even after adjusting for number of state officers per capita (ρ=0.85).

Note: DC’s data includes only 2016-2022, as it was not included in 2013 LEMAS data. DC’s data for 2013-2015 was thus excluded from this analysis.

**Supplemental Figure 5**. State-level rates of deaths attributable to local law enforcement, generally vs. per officer, 2013-2022

Here we compare rates of fatal violence by local/county law enforcement officers (including sheriffs) at the state level (along the X axis) by the same rates divided by the number of local/county law enforcement officers employed by a given state (along the Y axis). The linear pattern demonstrates that states that experience high levels of violence by local law enforcement officers in general also tended to have high levels of that violence even after accounting for the fact that those states tend to employ a larger number of local law enforcement officers, though the correlation was less pronounced here than it was for state officers (ρ=0.23).

Note: DC’s data includes only 2016-2022, as it was not included in 2013 LEMAS data. DC’s data for 2013-2015 was thus excluded from this analysis.

**Supplemental Figure 6**. Modeled state-level residuals from models estimating rates of deaths attributable to local law enforcement, adjusting vs. not adjusting for number of local officers per capita, 2013-2022

Here we compare state-level residuals from multilevel Poisson models estimating rates of fatal violence by local law enforcement officers at the state level, from a null model (along the X axis) vs. a model adjusting for the number of local law enforcement officers per capita in each state (along the Y axis). States that experienced high levels of violence by local law enforcement officers in general also tended to have high levels of that violence even after adjusting for number of local officers per capita (ρ=0.98).

Note: DC’s data includes only 2016-2022, as it was not included in 2013 LEMAS data. DC’s data for 2013-2015 was thus excluded from this analysis.
